# Impact of Vaccination on Rotavirus Genotype Diversity: A Nearly Two-Decade-Long Epidemiological Study before and after Rotavirus Vaccine Introduction in Sicily, Italy

**DOI:** 10.3390/pathogens11040424

**Published:** 2022-03-31

**Authors:** Floriana Bonura, Leonardo Mangiaracina, Chiara Filizzolo, Celestino Bonura, Vito Martella, Max Ciarlet, Giovanni M. Giammanco, Simona De Grazia

**Affiliations:** 1 Department of Health, Promotion, Mother and Child Care, Internal Medicine and Medical Specialties, University of Palermo, Piazza delle Cliniche 2, 90127 Palermo, Italy; floriana.bonura82@gmail.com (F.B.); leonardo.mangiaracina24@gmail.com (L.M.); chiarafilizzolo@libero.it (C.F.); celestino.bonura@unipa.it (C.B.); giovanni.giammanco@unipa.it (G.M.G.); 2 Department of Veterinary Medicine, University of Bari Aldo Moro, 70010 Valenzano, Italy; vito.martella@uniba.it; 3 Clinical Development, Icosavax, Seattle, WA 98102, USA; max.ciarlet@icosavax.com

**Keywords:** rotavirus, genotypes, vaccine, Rotarix, antigenic epitopes

## Abstract

Sicily was the first Italian region to introduce rotavirus (RV) vaccination with the monovalent G1P[8] vaccine Rotarix^®^ in May 2012. In this study, the seasonal distribution and molecular characterization of RV strains detected over 19 years were compared to understand the effect of Rotarix^®^ on the evolutionary dynamics of human RVs. A total of 7846 stool samples collected from children < 5 years of age, hospitalized with acute gastroenteritis, were tested for RV detection and genotyping. Since 2013, vaccine coverage has progressively increased, while the RV prevalence decreased from 36.1% to 13.3% with a loss of seasonality. The local distribution of RV genotypes changed over the time possibly due to vaccine introduction, with a drastic reduction in G1P[8] strains replaced by common and novel emerging RV strains, such as equine-like G3P[8] in the 2018–2019 season. Comparison of VP7 and VP4 amino acid (aa) sequences with the cognate genes of Rotarix^®^ and RotaTeq^®^ vaccine strains showed specific aa changes in the antigenic epitopes of VP7 and of the VP8* portion of VP4 of the Italian RV strains. Molecular epidemiological surveillance data are required to monitor the emergence of novel RV strains and ascertain if these strains may affect the efficacy of RV vaccines.

## 1. Introduction

Before the introduction of rotavirus (RV) vaccines, RV acute gastroenteritis (AGE) in infants and children under five years of age led to approximately 500,000 deaths each year, mainly in developing countries, where the access to safe drinking water and sanitary care are sub-optimal. With the introduction of RV vaccines, the current annual mortality estimates in children under five years of age range from 122,322 to 21,575, with deaths still mainly occurring in developing countries [1]. In developed countries before RV vaccine introduction, RV infections resulted in a substantial economic impact on healthcare systems and families due to medical visits, emergency room access and hospitalizations [2,3]. In 2006 and 2008, two oral RV vaccines, a pentavalent human-bovine reassortant vaccine (RotaTeq^®^ [Merck & Co] West Point, PA, USA, RV5) and a monovalent vaccine (Rotarix^®^ [GSK], Rixensart, Belgium, RV1), were licensed [4]. Both vaccines demonstrated high efficacy (>85%) against severe AGE episodes [5,6]. To date, almost 100 countries have introduced RV vaccines, including countries in Sub-Saharan Africa, the Americas, Europe, and the Eastern Mediterranean/Middle East region (The RV Organization of Technical Allies (ROTA) Council. http://rotacouncil.org/vaccine-introduction/global-introduction-status/ accessed on 7 July 2021). The impact on severe RV and all-cause diarrhea has been notable in countries that have introduced the vaccine. Epidemiological surveys on RV prevalence and molecular characterization of the viral strains circulating in the pediatric population are important to monitor vaccine effectiveness and to promptly detect novel RV strains/variants that might emerge under vaccine pressure and potentially affect vaccine-acquired protection.

RVs are non-enveloped viruses with a complex architecture. RV particles consist of three concentric capsid layers surrounding eleven segments of double-stranded RNA, which encode six structural proteins (VP1–VP4, VP6, and VP7) and six non-structural proteins (NSP1–NSP6) [7]. The outer protein layer is composed of VP4 and VP7 proteins, which independently elicit neutralizing antibodies and induce protective immunity [3,8]. A binary classification of RVs has been developed based on VP7 and VP4 genes, encoding, respectively, for a glycoprotein (G-genotype) and protease-sensible protein (P-genotype) [9]. RVs evolve through accumulation of point mutations, rearrangement, reassortment, and interspecies transmission [10,11], generating a heterogeneous population of viruses in continuous evolution. Currently, 41 G-genotypes and 57 P-genotypes have been reported in humans and animals, and more than 100 G/P combinations occur in nature (rega.kuleuven.be/cev/viralmetagenomics/virus-classification/rcwg). However, the majority of medically important human RVs belong to six G/P combinations (G1P[8], G2P[4], G3P[8], G4P[8], G9P[8], and G12P[8]) [12,13]. 

Sicily was the first Italian region to introduce RV vaccination into the routine childhood immunization schedule (June 2012). Since then, Rotarix^®^ (monovalent G1P[8] (GlaxoSmithKline [GSK]Vaccines, Rixensart, Belgium) has been the vaccine offered by the local healthcare authority, being administered on a two-dose schedule at 3 and 7 months of age. Vaccine coverage in 2012 was very low (6%) in Sicily but progressively increased from 20% in 2013 to 54% in 2019. In countries implementing universal RV vaccination, surveillance data have shown that vaccine introduction caused a reduction in RV disease burden, but also an increase in selective pressure exerted on strains circulating into human populations [14,15,16].

In Palermo, Sicily, more than 30 years (uninterruptedly since 1985) of RV surveillance data are available. This represented a unique opportunity to monitor changes in the prevalence of RV genotypes and to assess temporal patterns of variation over a large time window and a broad number of samples. This remarkable epidemiological setting provides a robust context through which to evaluate the local effects of the introduction of RV vaccination.

In this study, we compared the incidence of severe RV AGE, the seasonal distribution of RV genotypes, and the molecular epidemiological data of RVs causing infections in Sicily before (January 2002 to December 2012) and after (January 2013 to December 2020) the introduction of the RV vaccine.

## 2. Results

### 2.1. Seasonality and G/P Temporal Distribution

In total, 7846 samples collected during 19 consecutive years of hospital-based surveillance, from January 2002 to December 2020, were retrospectively analyzed to evaluate the epidemiological changes in RV ecology after the introduction of the monovalent G1P[8] vaccine (Rotarix^®^, RV1) in Sicily, Italy. The overall rate of RV-positive samples detected during the entire study period was 22.6%. Out of the 1773 RV-positive samples detected over the whole study period, 1157 (65.2%) were detected from 2002 to 2012, and 616 (34.7%) from 2013 to 2020. All the RV-positive samples were used for epidemiological analyses of the pre- and post-vaccine era, respectively (Table 1). 

**Figure 1:**
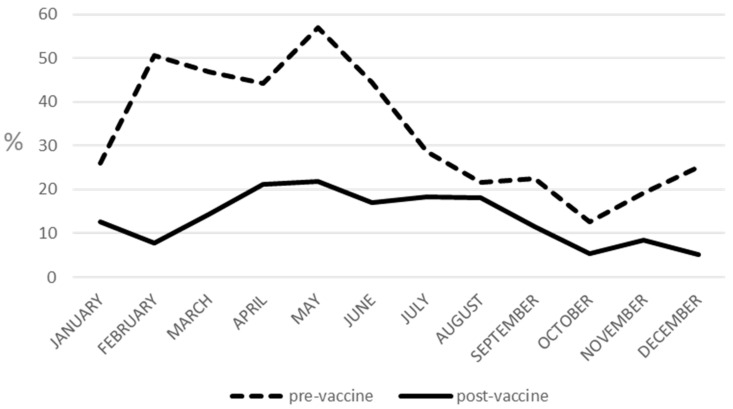
Comparison of rotavirus infection monthly prevalence during pre- (2002–2012) and post-vaccine (2013–2020) periods.

RV infection yearly prevalence rates ranged from 15.54% to 54.21% in the pre-vaccine period, with a combined pre-vaccine prevalence of 36.18%, and from 6.20% to 26.59% after vaccine introduction, with a combined prevalence of 13.36% over the post-vaccine period. A significant difference in pre- and post-vaccine periods was observed when comparing the yearly prevalence of RV infection in the two study periods (two-sample *t*-test; *p* < 0.05). In pre-vaccine era, from 2002 to 2012, RV infection exhibited increased seasonal activity from the end of winter to the end of spring, with prevalence ranging from 56.96% to 44.24% from February to May. While in the post-vaccine era, from 2013 to 2020, no period of specially increased circulation could be observed; only a plateau of prevalence rates above or approaching 20% (range: 16.98–21.83%) extending from the end of spring to summer was observed (Figure 1). Interestingly, when comparing data from pre- and post-vaccine periods, a 1.2- to 6.5-fold decline in monthly RV infection rates was observed after vaccine introduction (Table 2). 

Among the 1773 RV-positive samples detected during the study period, an RV genotype was determined for 1651 (93.1%) samples. Specifically, 1082 out of 1157 (93.5%) RV-positive specimens were genotyped in the pre-vaccine period and 569 out of 616 (92.37%) in the post-vaccine period.

**Figure 2:**
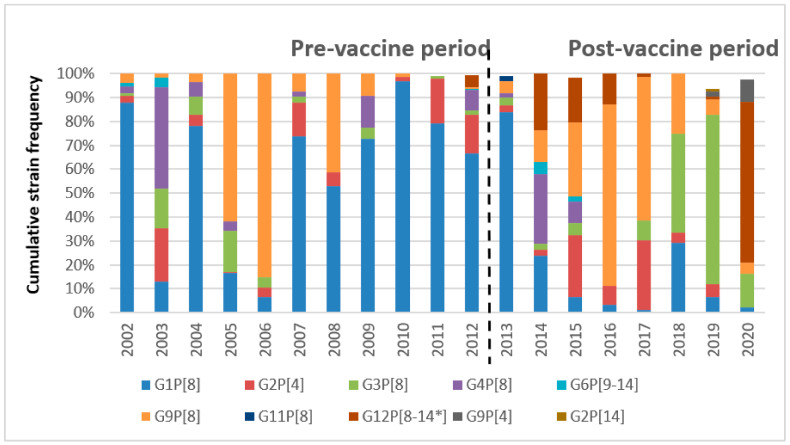
Cumulative rotavirus genotypes distribution over the pre-vaccine (2002–2012) and post-vaccine (2013–2020) periods. * The G12 RV was detect in association with P[8] or P[14] genotype.

From 2002 to 2012, G1P[8] was the prevalent RV genotype, accounting for 62.4% of the cases, followed by G9P[8] (18.39%), G2P[4] (9.35%), G4P[8] (6.61%), G3P[8] (5.28%), and G12P[8] (1.12%). In the pre-vaccine period, increased circulation of G4P[8] (42.6%) in 2003 and G9P[8] (61.6–84.5%) in 2005-06 exceeded the otherwise yearly prevalent G1P[8] strains. In the post-vaccine period, G9P[8] exceeded G1P[8] strains, being responsible for 29.17% of RV infections, versus 20.56% due to G1P[8]. G2P[4] represented the third most common genotype (12.65%), followed by G3P[8] (17.40%), G12P[8] (12.48% ), and G4P[8] (4.22%). Other genotypes, such as G6, G8, G10, and G11, were detected sporadically, accounting for less than 1% of RV infections over the study period. The scenario of temporal genotypes’ distribution changed over the time after the introduction of RV vaccine. G1P[8] drastically decreased from 2014 to 2020 (from 23.68 to 1.16%), while G9P[8] predominated between 2016 (70.19%) and 2017 (60.47%), with G3P[8] becoming the prevalent genotype in 2018 onwards, reaching a prevalence of 70.97% in 2019, and G12P[8] strains emerging in 2012 (5.34%) to prevail in 2020 (67.44%). A fluctuating circulation of G2P[4] and a relatively low prevalence of G4P[8] RVs were observed over the study period (Figure 2). 

Over the entire study period, the VP4 P[8]-genotype represented the predominant P-type (85.77%), followed by P[4] (12.92%), while P[6], P[9], and P[14] were detected only sporadically (<1%). A low prevalence (<1%) of mixed infections involving the P[8]-genotype and other P-types was also observed.

### 2.2. VP7 and VP4 Phylogenetic Analyses

**Figure 3:**
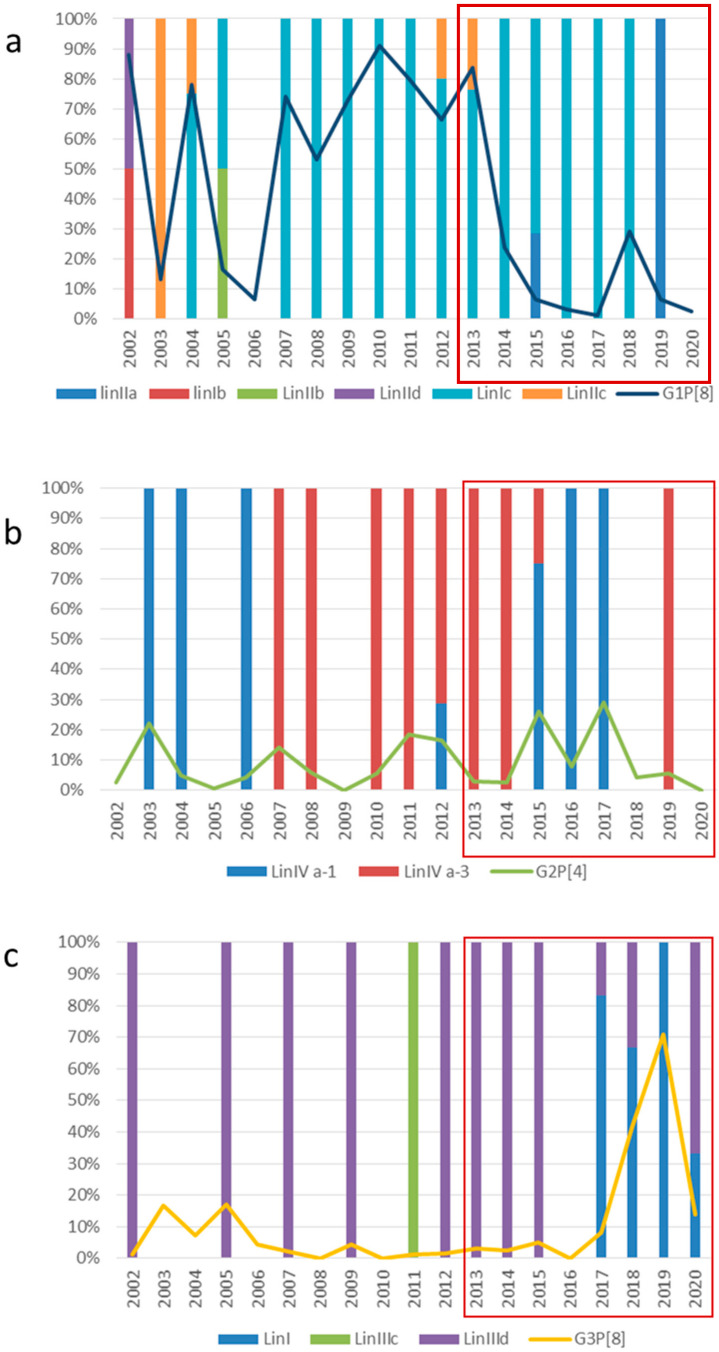

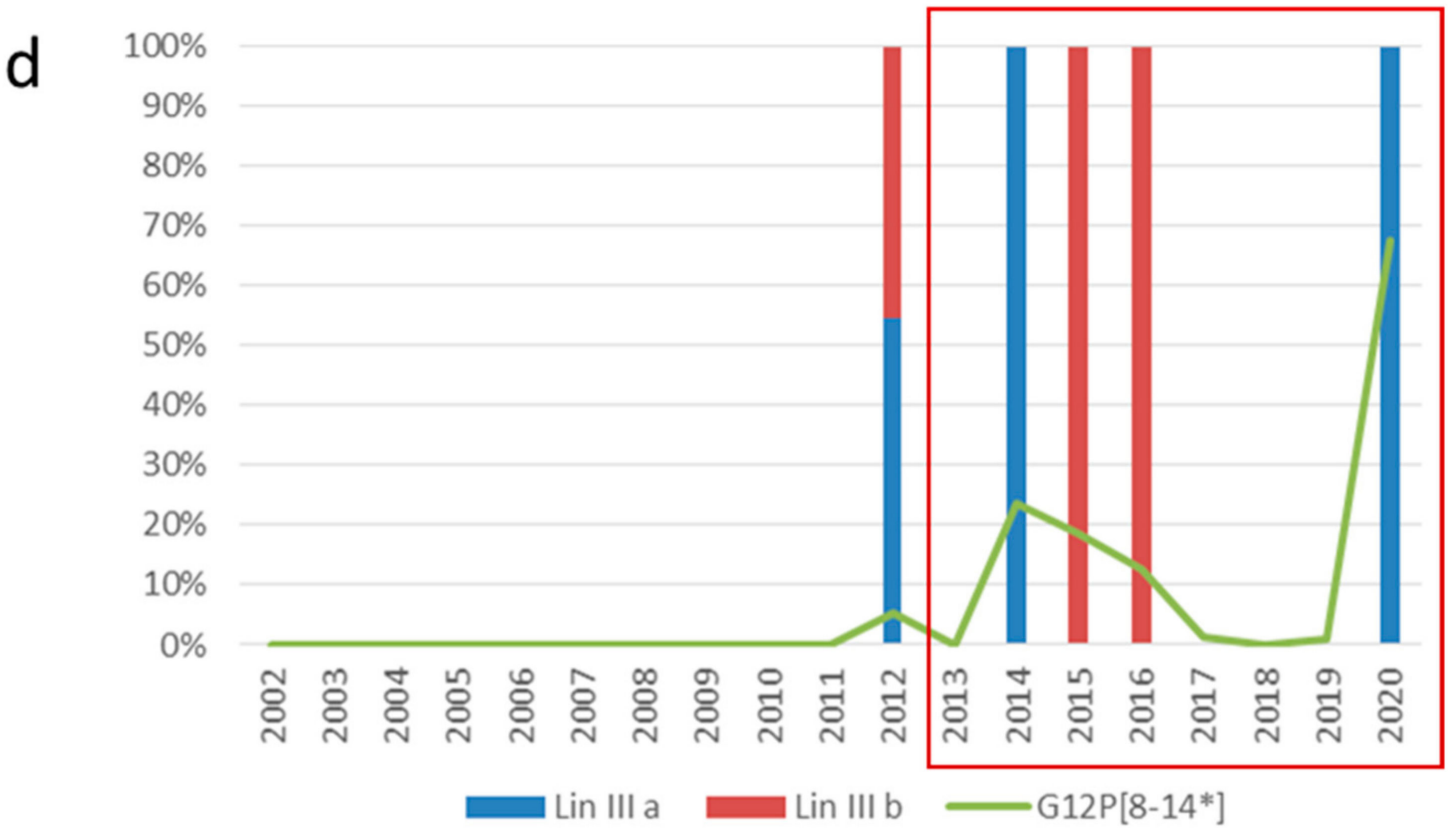
Temporal distribution over the study period of VP7 lineages and sublineages of Rotavirus G1P[8] (**a**), G2P[4] (**b**), G3P[8] (**c**), and G12P[8] (**d**). Overlaid lines show the prevalence rate of each genotype and the post-vaccine period (2013–2020) is highlighted in the box. * The G12 RV was detect in association with P[8] or P[14] genotype.

For a selection of RVs belonging to different G- and P-types, the VP7 and VP4 genes were sequenced and analyzed phylogenetically (Supplementary Figure S1). Among the G1P[8] strains, a single sub-lineage, Ic, which had been circulating since 2004, was predominant (75% of the sequenced strains) until 2019, when it was replaced by sub-lineage IIa (Figure 3a). In the VP7 gene, the Italian G1-Ic RVs showed a percentage of nucleotide (nt) and amino acid (aa) identity ranging from 95 to 100% similarity to each other and from 91 to 95% nt and 91 to 97% aa similarity to the Rotarix^®^ strain (sub-lineage IIa), and ranging from 89 to 91% nt and from 90 to 94% aa similarity to the RotaTeq^®^ strain (lineage III). All the G1-IIa strains detected were vaccine strains showing a 98.3–100% nt and 98–100% aa identity similarity to the Rotarix^®^ strain. The Italian G2P[4] RVs detected from 2003 to 2019 clustered into lineage IV, in two distinct sub-lineages, a-1 and a-3, alternating over time (Figure 3b). The two sub-lineages showed an intra sub-lineage, nt, and aa identity of 96 to 100% and an inter sub-lineage nt and aa identity of 91–97% and 96–99%, respectively. The VP7 of the RotaTeq^®^ G2 strain clustered into lineage II and showed a 92–94% nt and 92–97% aa identity similarity to the Italian G2-a-1 strains and a 91–94% of nt and 91–99% aa identity similarity to GII-a-3 strains. Among the G3P[8] RVs, the Italian strains detected from 2004 to 2018 clustered in lineage III together with “classical” human strains (96.5–100% nt identity and 95.8–100% aa identity), while the G3P[8] strains that had been detected since 2019 segregated into lineage I together with novel equine-like RVs (98.8–100% nt identity and 97.1–100% aa identity). A contemporaneous circulation of G3P[8] equine-like strains, clustering into lineage I (98–100% nt and a 96–100% aa identity), and “classical” G3P[8] strains of lineage III (97–100% nt and a 98–100% aa identity) was observed in 2020 (Figure 3c). 

Sequence analyses of the VP7 gene of G4P[8] RVs showed that all Italian strains detected from 2002 to 2020 fell together into lineage Ic, with a 98–100% nt and a 96–100% aa similarity to each other. These G4 strains showed a 94–96% nt and 86–90% aa identity similarity to the RotaTeq^®^ G4 strain. G9P[8] RVs were frequently detected in both the pre- and post-vaccine periods, and they all belonged to a major sub-cluster within lineage III (94–100% nt and 90–100% aa identity). All the Italian G12P[8] RVs clustered into lineage III, but into two different sub-lineages (III-a and III-b). In particular, the G12 strains detected in 2012 and 2014 were equally distributed into sub-lineages III-a and III-b, whilst all 2015 and 2016 G12 strains clustered in sub-lineage III-b, and the 2020 G12 strains were segregated into sub-lineage III-a. Within these sub-lineages, the identity was 96–100% nt and 98–100% aa for lineage III-a and 95–100% nt and 95–100% aa for lineage III-b (Figure 3d). 

In the VP8* portion of VP4, all the VP4 P[8] strains, regardless of the associated G-type (G1, G3, G4, G9, and G12), clustered into lineage III, showing 92 to 100% similarity in nt and aa identities to each other. When compared to the vaccine strains, the P[8] strains showed 87 to 91% nt identity and 86 to 92% aa identity similarity to the Rotarix^®^ strain (Lineage I), and 89–94% nt and 88–96% aa identity similarity to RotaTeq^®^ strain (lineage II). The Italian P[4] strains clustered into lineage IV (93–100% of nt and 91–100% aa identities). Phylogenetic analysis showed a lower genetic diversity without any apparent temporal pattern of distribution among P[4] RVs.

### 2.3. Comparative Analysis of Neutralizing Epitopes of the VP7 and VP4 Proteins 

The deduced amino acid sequences of neutralization epitopes (7-1a, 7-1b and 7-2) of VP7 and (8-1 and 8-2) of the VP8* portion of VP4 of the Italian RV strains were compared with homologous sequences of the Rotarix^®^ and RotaTeq^®^ vaccine strains, allowing for the identification of peculiar amino acid substitutions. In particular, all the Italian G1 strains of sub-lineage Ic, detected both in the pre- and post-vaccine periods, showed four mismatches when compared to Rotarix^®^ (A1CB052A/1988/G1P[8]) and six compared to RotaTeq^®^ (WI79-9/1992/G1P7[5]). All G1 RVs of sub-lineage IIa shared the same amino acid sequence with Rotarix^®^, except for a unique mutation (S123N) observed in two different 2015 strains (Table 3a). The Italian G2 strains of lineage IVa-1 and IVa-3 differed in three aa residues from RotaTeq^®^ (SC2-9/1992/G2P7[5]), in epitopes 7-1a and 7-1b. An additional VP7 substitution S96N was observed only in 2015 G2 strains (Table 3b). The classical Italian G3P[8] strains (lineage I) showed three mismatches with respect to RotaTeq^®^ (WI78-8/1992/G3P75) in the 7-1b neutralizing epitope, with an additional change (T87I) observed for three 2018 G3 strains in the 7-1a epitope, while the equine-like G3 strains differed from the vaccine VP7 in four residues (T87S in 7-1a epitope and N213T, K238D and D242A in 7-1b epitope). All the 2019 G3 strains showed an additional substitution in the 7-1b epitope (A212T) (Table 3c). The G4P[8] Italian strains showed four conserved aa substitutions with respect to the RotaTeq^®^ strain (BrB-9/1996/G4Ia) (Table 3d).

Upon analysis of the VP8* proteolytic cleavage product of the VP4 protein, specific aa substitutions were identified in the 8-1 and 8-3 antigenic epitopes comparing the Italian P[8] strains and Rotarix^®^ (A1CB052A/1988/G1P[8]) and RotaTeq^®^ (WI79-4/1992/G6P[8]) vaccine strains. The Italian P[8]-I lineage strains shared conserved amino acid sequences with Rotarix^®^, with the exception of a change (K/D113N) in the 8-3 epitope. All P[8]-III strains differed from both vaccine strains at two aa positions (F150D and N195G) in the 8-1 epitope and showed three addition substitutions (S125N, S131R, and N135D) in the 8-3 epitope with respect to Rotarix^®^ strain. Two sporadic mutations, N194D in epitope 8-1 and N113D in epitope 8-3, were observed in the majority of P[8]-III strains, including all equine-like G3 RVs (Table 4).

## 3. Discussion

Then RV vaccine was introduced in Sicily, Italy, in June 2012. The present study investigated the epidemiology of RV infection before (from 2002 to 2012) and after (from 2013 to 2020) vaccine introduction. Based on the epidemiological data, the increasing vaccine coverage induced a marked decrease in RV infection prevalence from 2013 onward, with an overall prevalence rate declining from 36.18% in the pre-vaccine period to 13.36% in the post-vaccine period. Moreover, we observed the loss of the previously strong winter/spring seasonality of RV infection, with a remarkable decrease (ranging from 1.2 to 6.5-fold) in RV infections during winter and spring in the post-vaccine period (Figure 1, Table 2). Typically, RV infection exhibits peaks of seasonal activity during the winter season in temperate climates and throughout the year in tropical climates [17]. A delay and a blunting of seasonal peaks were also observed in other temperate climate countries where vaccination had been introduced, compared to the pre-vaccine period [18,19,20,21,22]. 

Based on the epidemiological information generated in our hospital-based surveillance, changes in the distribution of RV genotypes in Italy occurred after the introduction of RV vaccination. In particular, the previously predominant G1P[8] genotype, which accounted for more than 60% of RV infections in the pre-vaccine period, drastically decreased from 2014 to 2020. This strain was replaced by different genotypes in the post-vaccine period. A marked genotype diversity was observed in 2014–2015 when G1P[8] strains co-circulated at low prevalence with several other genotypes, i.e., G2P[4], G4P[8], G9P[8], and G12P[8]. The predominance of G1P[8] RV prior to vaccine introduction has been thoroughly described, as well as fluctuations in RV genotypes distribution and a switch in predominant genotypes, from G1P[8] to G2P[4] following Rotarix^®^ introduction [4,23,24,25,26].

In the present study, in the post-vaccine era, the alternate prevalence of a variety of RV genotypes was observed, including G2P[4] in 2015 and 2017, G9P[8] in 2016–2017, G3P[8] in 2018–2019, and G12P[8] in 2020 (Figure 2). The emergence of these genotypes could have been driven by the force of vaccine selection, which can impose a selective pressure on circulating strains or by a selection of mutant viruses that are not effectively neutralized [4]. Alternatively, this could be the effect of natural seasonal fluctuations and global emergence of novel strains generated by natural variation or re-assortment events between human and animal strains [27,28]. In Palermo, RV surveillance activity spanned more than three decades, and the genotypes circulating in our relatively small geographic area mostly reflected the epidemiological scenario observed in Europe and in most developed countries [29,30]. Novel genotypes, such as G9P[8] in 1999–2000 and G12P[8] in 2012, emerged in Italy, acquiring epidemiological relevance over time [31,32]. Recently, novel equine-like G3P[8] strains, likely deriving from a reassortment event with equine RV strains, spread in several countries, becoming the prevalent genotype in Italy in 2018–2019 [33]. Interestingly, the circulation of equine-like G3P[8] strains abruptly dropped in 2020, replaced by G12 RV strains. Similar fluctuations of prevalent RV genotypes, with G1P[8] being replaced first by G9P[8] and more recently by G12P[8] and G3P[8] genotypes, were also observed in Brazil after the introduction of universal vaccination [34]. Several studies showed a cyclic rise in detection rates of non-G1P[8] strains in both vaccinated and non-vaccinated children [28,30,34,35]. Unfortunately, the vaccination status of the children enrolled in this study was not available and it was not possible to evaluate the differential circulation of RV types in vaccinated and non-vaccinated groups. 

Genetic drift has generated several lineages and sub- lineages within RV genotypes, providing some strains with increased fitness or the ability to escape partially population immunity, and this could be related to the epidemic peaks of RV infections [15]. In previous studies, it was hypothesized that G1P[8] continuous circulation was due to the emergence end re-emergence of different lineages and sub-lineages [36]. Sub-lineage Ic, only sporadically detected in 1996–1997, 2002, and 2004, was predominant among G1P[8] strains after 2007 in Italy [29]. In this study, G1 RVs of sub-lineage Ic were found to circulate both in the pre- and post-vaccine period. A marked intra-genotype diversity, with different lineages replacing each other, was observed within G2P[4], G3P[8] and G12P[8] genotypes (Figure 3), while a single lineage of G9P[8] (lineage III) and G4P[8] (lineage Ic) RVs was detected. 

The genetic diversity observed in the VP8* portion of the VP4 was lower, probably due to structural and functional constraints during attachment, penetration, and maturation of the virion. In the present study, a single VP4 lineage (P[8]-III) was observed in all P[8] strains detected over the entire study period, regardless of the associated G-type. As an exception to this rule, the G1P[8] vaccine-derived strains segregated into lineage P[8]-I. 

Upon comparison of VP7 and VP4 aa sequences with the cognate genes of Rotarix^®^ and RotaTeq^®^ vaccine strains, the Italian RVs showed specific aa changes in the antigenic epitopes of VP7 (7-1a, 7-1b, and 7-2) and of the VP8* portion of VP4 (8-1 to 8-4). 

The VP7 sequences of Italian G1-Ic RVs, both in the pre- and post-vaccine period, showed up to four and six conserved aa substitutions with respect to Rotarix^®^ (A1CB052A/1988/G1P[8]) and RotaTeq^®^ (WI79-9/1992/G1P7[5]), respectively (Table 3a). Changes at residues 94, 123, and 217 are correlated with neutralization resistance and have been also observed in G1P[8] strains circulating in several countries [37,38]. Peculiar amino acid changes, mainly located in the 7-1a and 7-b epitopes of VP7, were detected in all G2 and G4 Italian strains circulating over the entire study period. Some VP7 changes (A87T, D96N, and S213D) have been associated with the emergence of G2P[4] in different countries, following vaccine introduction, possibly due to differential antigenic pressure triggered by the monovalent G1 vaccine [15,27]. However, in our study, G2P[4] strains did not acquire particular relevance after vaccine introduction. On the other hand, a significant epidemiological change was observed in Italy in the G3P[8] genotype, with the emergence of equine-like G3 strains in 2017. Classical and equine-like G3P[8] strains showed peculiar patterns of aa substitutions with respect to the sequence of RotaTeq^®^ vaccine (Table 3d). Conserved aa changes, mainly located in the 8-1 and 8-3 epitopes of VP4 protein, were observed in Italian P[8] strains of lineage III, with respect to Rotarix^®^ (A1CB052A/1988/G1P[8]) and RotaTeq^®^ (WI79-4/1992/G6P[8]) vaccine strains of lineage I and II, respectively (Table 4). Such substitutions were peculiar of the P[8]-III lineage, which represents the most common VP4 type in globally circulating RVs [39]. 

The major antigenic changes observed in equine-like G3P[8] might allow these strains to successfully spread globally in a relatively short period [38]. The identification of notable aa substitutions in each genotype detected in both pre- and post-vaccine era might be responsible for a fitness advantage, which allowed such rotaviruses to maintain their circulation without being affected by vaccines. Otherwise, as a result of immunization strategies, the decreased circulation of RVs among humans may have reduced their innate tendency to mutate, as observed for circulating vaccine-derived polioviruses, which preferentially mutate and cause the disease in communities with low immunization rates (https://polioeradication.org/polio-today/polio-now/this-week/circulating-vaccine-derived-poliovirus/ accessed on 25 February 2022).

## 4. Material and Methods

### 4.1. Study Population

To evaluate the RV variability in pre- and post-vaccine era, data collected in eight consecutive years after vaccine introduction (from January 2013 to December 2020) were compared to those collected in the 10 years before vaccine introduction (from January 2002 to December 2012). Pre-vaccine period included the entire year 2012 since vaccination was introduced only in June and coverage was considered to be too low (6%) to affect RV circulation. Overall, a total of 7846 children under 5 years of age, hospitalized with AGE at the ‘‘G. Di Cristina’’ Children’s Hospital of Palermo, Italy, between 2002 and 2012 (pre-vaccination period, *n* = 3213) and from 2013 to 2020 (post-vaccination period, *n* = 4633), were enrolled (Table 1). This study is a retrospective study using hospital-based surveillance data and represents an extension and integration of a previous study, summarizing the results of a 27-year-long RV surveillance period in the pre-vaccination period [29].

### 4.2. G and P Genotyping and Phylogenetic Analyses

All stool samples were subjected to RNA extraction (QIAamp Viral RNA MiniKit, Qiagen, Hilden, Germany) and analyzed using random primers reverse transcription shortly after samples were collected [40] and real-time PCR with specific primers targeting the NSP3 gene of RV as described previously [41]. Specimens in which RV RNA was detected at a cycle threshold (Ct) value ≤ 30 were considered positive and were eligible for molecular characterization. RV-positive specimens (1495) were further analyzed to determine the G/P genotypes, according to the established binomial classification, using a mixture of specific primers (genotypes G1-G4, G6, G8, and G9-G12 and P[4], P[6], P[8]-P[11], and P[14], for VP7 and VP4 types, respectively) [42,43,44]. 

In addition, RV-positive fecal samples with sufficient quantity, representative of the entire study period, were submitted to sequence analyses. The nearly full-length VP7 gene and the VP8* portion of the VP4 gene were amplified with consensus primers and the sequences were determined by direct sequencing. Sequence alignment was performed with CLUSTAL W [45]. Phylogenetic analysis was carried out using the MEGA software version X [46] using the Kimura 2-parameter model as a method of substitution and the maximum likelihood method to construct phylogenetic trees from partial sequences of VP7 and VP4. Sequences were assigned to lineages/sulineages as described in previous literature [29]. Representative sequences of RVA strains belonging to different genotypes, used to generate phylogenetic trees, have been deposited in GenBank.

### 4.3. Statistical Analyses

The yearly pre- and post-vaccine prevalence was calculated as rotavirus-positive samples/samples tested, and a two-sample *t*-test was used to compare the yearly prevalence rates recorded in each of the two study periods. A *p*-value ≤ 0.05 was considered statistically significant. All the analyses were performed with http://vassarstats.net, accessed on 15 January 2022.

## 5. Conclusions

The introduction of anti-RV universal vaccination in Sicily encouraged monitoring of its immune-epidemiological effects. Comparative analysis of RVs detected before and after the introduction of vaccination, besides a marked reduction in the prevalence of infection, also showed increased genetic diversity in RVs. The post-vaccine emergence of G3P[8] equine-like strains and the increasing circulation of G12P[8] RVs highlight the need for molecular epidemiological surveillance in order to promptly reveal the emergence of novel RV strains, and eventually ascertain if these RV variants may escape vaccine-induced immunity.

## Figures and Tables

**Table 1:** Prevalence of rotavirus infection in pre- (2002–2012) and post-vaccine (2013–2020) study periods.

	Year of Surveillance	No. of Samples Tested	No. of Rotavirus-Positive Samples	Rotavirus Prevalence (%)
Pre-vaccine period	2002	196	76	38.78
	2003	214	55	25.70
	2004	196	82	41.84
	2005	273	148	54.21
	2006	179	47	26.26
	2007	217	93	42.86
	2008	154	68	44.16
	2009	342	67	19.59
	2010	386	60	15.54
	2011	377	126	33.42
	2012	679	237	34.90
	Overall Pre-vaccine	3.213	1.059	36.18
Post-vaccine period	2013	759	119	15.68
	2014	627	47	7.50
	2015	504	134	26.59
	2016	492	64	13.01
	2017	707	87	12.31
	2018	468	29	6.20
	2019	683	93	10.94
	2020	393	43	25.44
	Overall Post-vaccine	4.633	616	13.36

**Table 2:** Rotavirus infection monthly prevalence in pre-vaccine (2002–2012) and post-vaccine (2013–2020) era. Seasonal peaks of circulation are shown in grey.

Months	Pre-Vaccine Prevalence (%)	Post-Vaccine Prevalence (%)
**January**	26.07	12.59
**February**	50.55	7.75
**March**	46.99	14.48
**April**	44.24	21.21
**May**	56.96	21.83
**June**	44.51	16.98
**July**	28.57	18.25
**August**	21.71	18.08
**September**	22.41	11.47
**October**	12.60	5.43
**November**	19.18	8.51
**December**	25.22	5.09

**Table 3:** Amino acid (aa) substitutions in neutralizing epitopes of VP7 (7-1a, 7-1b, and 7-2) observed in Italian RV strains: (a) G1P[8], (b) G2P[4], (c) G3P[8], (d) and G4P[8], compared to vaccine strains (Rotarix^®^ and/or RotaTeq^®^).

**(a) G1P[8]**	**Epitopes**					
	**7-1a**			**7-1b**	**7-2**	
**Strain/sub-lineage** **(No. of sequences analyzed)**	**Positions**					
	**94**	**212**	**97**	**123**	**147**	**217**
Rotarix^®^ (A41CB052A /1988/G1P1A[8])	N	V	E	S	N	M
RotaTeq^®^ (WI79-9/1992/G1P7[5])	N	V	D	S	S	M
G1P[8] sub-lineage Ic (54)	S	T	E	N	N	T
G1P[8] sub-lineage IIa (6)	N	V	E	S/N	N	M
**(b) G2P[4]**	**Epitopes**					
	**7-1a**				**7-1b**	
**Strain/sub-lineage** **(No. of sequences analyzed)**	**Positions**					
	**87**		**96**		**213**	
RotaTeq^®^ (SC2-9/1992/G2P7[5])	A		D		S	
G2P[4] sub-lineage IVa-1 (18)	T		N/S		D	
G2P[4] sub-lineage IVa-3 (18)	T		N		D	
**(c) G3P[8]**	**Epitopes**					
	**7-1a**		**7-1b**			
**Strain/sub-lineage** **(No. of sequences analyzed)**	**Positions**					
	**87**		**212**	**213**	**238**	**242**
RotaTeq^®^ (WI78-8/1992/G3P75)	T		A	N	K	D
G3P[8] human (24)	T/I		T	N	N	N
G3P[8] equine-like (39)	S		T/A	T	D	A
**(d) G4P[8]**	**Epitopes**					
	**7-1a**		**7-1b**	**7-2**		
**Strain/sub-lineage** **(No. of sequences analyzed)**	**Positions**					
	**130**		**211**	**143**		**145**
RotaTeq^®^ (BrB-9/1996/G4Ia)	D		D	R		A
G4P[8] sub-lineage Ic (14)	E		N	K		T

The letters represent the peculiar amino acid substitutions of neutralization epitopes (7-1a, 7-1b and 7-2) of VP7 compared with homologous sequences of the Rotarix^®^ and RotaTeq^®^ vaccine strains, were showed in Table 3.

**Table 4:** Amino acid substitutions observed in neutralizing epitopes in the VP8* portion of VP4 of Italian P[8] RV strains when compared to vaccine strains (Rotarix^®^ and RotaTeq^®^).

		Epitopes						
		8-1			8-3			
	Strain/Sub-Lineage (No. of Sequences Analyzed)	Positions						
		150	194	195	113	125	131	135
**Vaccine**	Rotarix^®^ (A1CB052A/1988/G1P[8])	F	N	N	N	S	S	N
	RotaTeq^®^ (WI79-4/1992/G6P[8])	F	N	D	N	N	R	D
**P[8]-I**	G1P[8] (4)	F	N	N	N/K/D	S	S	N
**P[8]-III**	G1P[8] (50)	D	N/D	G/D	N/D	N	R	D
	G3P[8] (19)	D	N/D	G	N/D	N	R	D
	G3P[8] Eq-like (30)	D	D	G	D	N/K	R	D
	G9P[8] (43)	D	D	G	N	N	R	D
	G12P[8] (21)	D	N/D	G	N/D	N	R	D

The letters represent the peculiar amino acid substitutions of neutralization epitopes in the VP8* portion of VP4 compared with homologous sequences of the Rotarix^®^ and RotaTeq^®^ vaccine strains, were showed in Table 4.

## Supplementary Materials

The following are available online at https://www.mdpi.com/article/10.3390/pathogens11040424/s1, Figure S1: Phylogenetic analysis of partial VP7 (G1-4, G9 and G12) and VP4 (P[4]–P[8]) nucleotide sequences of Italian strains detected in the study. The phylogenetic trees were built using the neighbor-joining method and Kimura’s two-parameter model, and bootstrapped with 1000 repetitions. Bootstrap values 75% are indicated. * Sequences availables in GenBank.
