# Supplementary material for: Impact of Vaccination on Rotavirus Genotype Diversity: A Nearly Two-Decade-Long Epidemiological Study before and after Rotavirus Vaccine Introduction in Sicily, Italy
Source: Pathogens. 2022 Mar 31;11(4):424. doi: 10.3390/pathogens11040424 (PMC9028787; doi:10.3390/pathogens11040424)
Supplement: Supplementary file 1 [file pathogens-11-00424-s001.zip › pathogens-1649169-supplementary.pdf]

G1

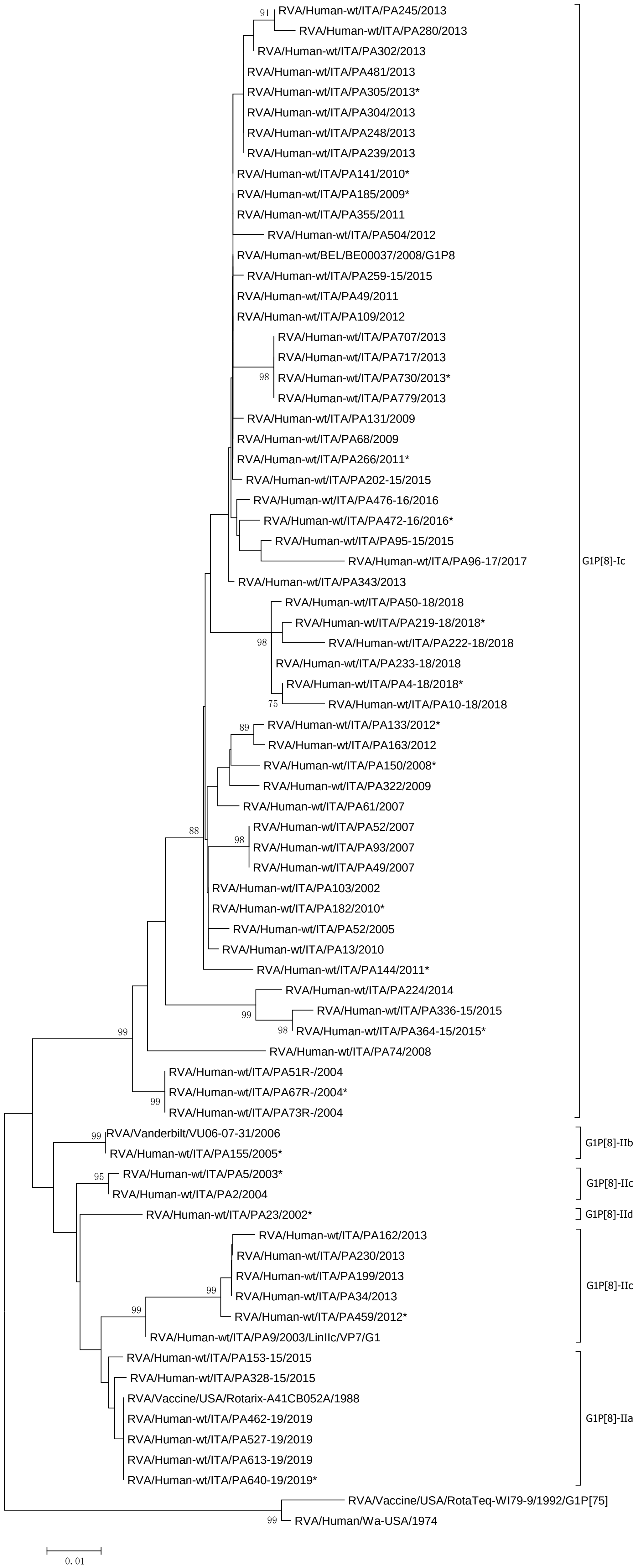
RVA/Human-wt/ITA/PA245/2013
RVA/Human-wt/ITA/PA280/2013
RVA/Human-wt/ITA/PA302/2013
RVA/Human-wt/ITA/PA481/2013
RVA/Human-wt/ITA/PA305/2013*
RVA/Human-wt/ITA/PA304/2013
RVA/Human-wt/ITA/PA248/2013
RVA/Human-wt/ITA/PA239/2013
RVA/Human-wt/ITA/PA141/2010*
RVA/Human-wt/ITA/PA185/2009*
RVA/Human-wt/ITA/PA355/2011
RVA/Human-wt/ITA/PA504/2012
RVA/Human-wt/BEL/BE00037/2008/G1P8
RVA/Human-wt/ITA/PA259-15/2015
RVA/Human-wt/ITA/PA49/2011
RVA/Human-wt/ITA/PA109/2012
RVA/Human-wt/ITA/PA707/2013
RVA/Human-wt/ITA/PA717/2013
RVA/Human-wt/ITA/PA730/2013*
RVA/Human-wt/ITA/PA779/2013
RVA/Human-wt/ITA/PA131/2009
RVA/Human-wt/ITA/PA68/2009
RVA/Human-wt/ITA/PA266/2011*
RVA/Human-wt/ITA/PA202-15/2015
RVA/Human-wt/ITA/PA476-16/2016
RVA/Human-wt/ITA/PA472-16/2016*
RVA/Human-wt/ITA/PA95-15/2015
RVA/Human-wt/ITA/PA96-17/2017
RVA/Human-wt/ITA/PA343/2013
RVA/Human-wt/ITA/PA50-18/2018
RVA/Human-wt/ITA/PA219-18/2018*
RVA/Human-wt/ITA/PA222-18/2018
RVA/Human-wt/ITA/PA233-18/2018
RVA/Human-wt/ITA/PA4-18/2018*
RVA/Human-wt/ITA/PA10-18/2018
RVA/Human-wt/ITA/PA133/2012*
RVA/Human-wt/ITA/PA163/2012
RVA/Human-wt/ITA/PA150/2008*
RVA/Human-wt/ITA/PA322/2009
RVA/Human-wt/ITA/PA61/2007
RVA/Human-wt/ITA/PA52/2007
RVA/Human-wt/ITA/PA93/2007
RVA/Human-wt/ITA/PA49/2007
RVA/Human-wt/ITA/PA103/2002
RVA/Human-wt/ITA/PA182/2010*
RVA/Human-wt/ITA/PA52/2005
RVA/Human-wt/ITA/PA13/2010
RVA/Human-wt/ITA/PA144/2011*
RVA/Human-wt/ITA/PA224/2014
RVA/Human-wt/ITA/PA336-15/2015
RVA/Human-wt/ITA/PA364-15/2015*
RVA/Human-wt/ITA/PA74/2008
RVA/Human-wt/ITA/PA51R-/2004
RVA/Human-wt/ITA/PA67R-/2004*
RVA/Human-wt/ITA/PA73R-/2004
G1P[8]-Ic
RVA/Vanderbilt/VU06-07-31/2006
RVA/Human-wt/ITA/PA155/2005*
G1P[8]-IIb
RVA/Human-wt/ITA/PA5/2003*
RVA/Human-wt/ITA/PA2/2004
G1P[8]-IIc
RVA/Human-wt/ITA/PA23/2002*
G1P[8]-IId
RVA/Human-wt/ITA/PA162/2013
RVA/Human-wt/ITA/PA230/2013
RVA/Human-wt/ITA/PA199/2013
RVA/Human-wt/ITA/PA34/2013
RVA/Human-wt/ITA/PA459/2012*
RVA/Human-wt/ITA/PA9/2003/LinIIc/VP7/G1
G1P[8]-IIc
RVA/Human-wt/ITA/PA153-15/2015
RVA/Human-wt/ITA/PA328-15/2015
RVA/Vaccine/USA/Rotarix-A41CB052A/1988
RVA/Human-wt/ITA/PA462-19/2019
RVA/Human-wt/ITA/PA527-19/2019
RVA/Human-wt/ITA/PA613-19/2019
RVA/Human-wt/ITA/PA640-19/2019*
G1P[8]-IIa
RVA/Vaccine/USA/RotaTeq-WI79-9/1992/G1P[75]
RVA/Human/Wa-USA/1974
0.01

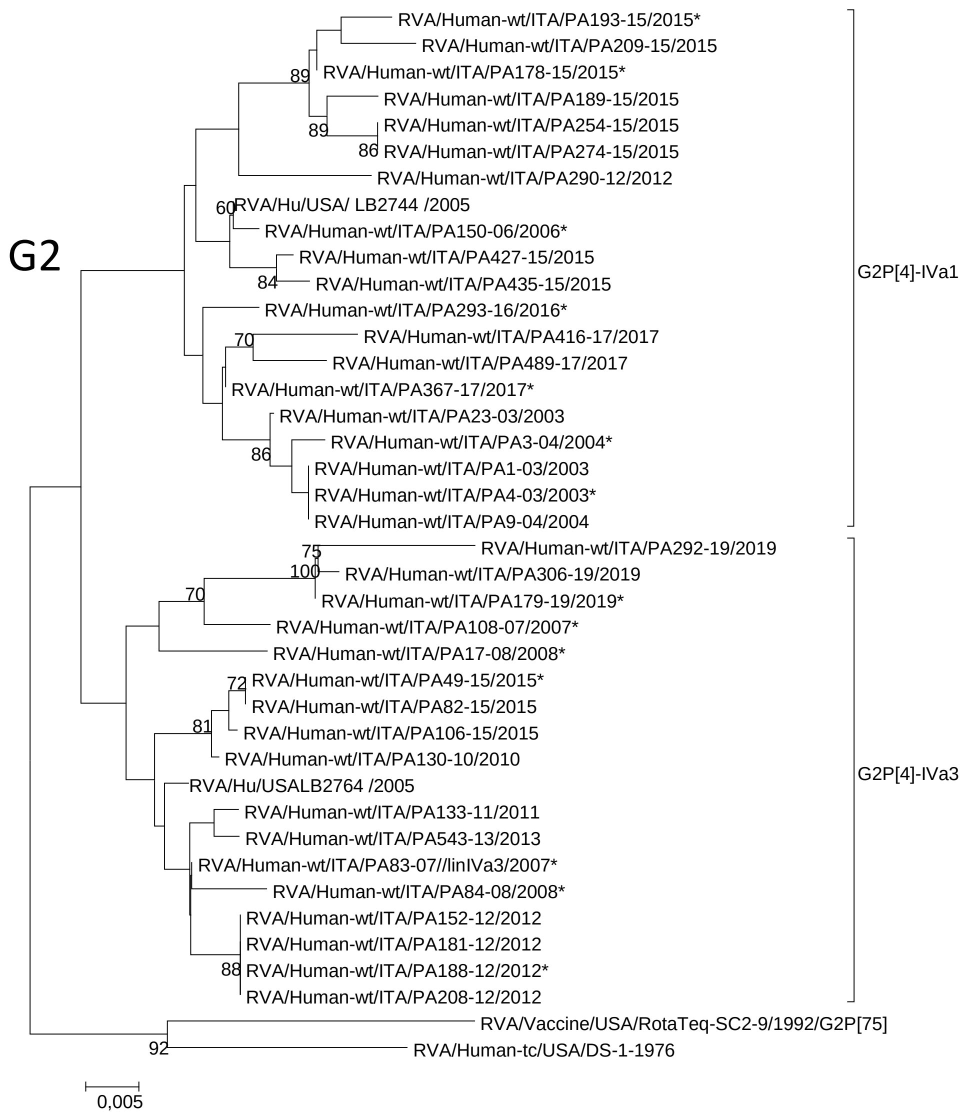
G2
RVA/Human-wt/ITA/PA193-15/2015*
RVA/Human-wt/ITA/PA209-15/2015
RVA/Human-wt/ITA/PA178-15/2015*
RVA/Human-wt/ITA/PA189-15/2015
RVA/Human-wt/ITA/PA254-15/2015
RVA/Human-wt/ITA/PA274-15/2015
RVA/Human-wt/ITA/PA290-12/2012
RVA/Hu/USA/ LB2744 /2005
RVA/Human-wt/ITA/PA150-06/2006*
RVA/Human-wt/ITA/PA427-15/2015
RVA/Human-wt/ITA/PA435-15/2015
RVA/Human-wt/ITA/PA293-16/2016*
RVA/Human-wt/ITA/PA416-17/2017
RVA/Human-wt/ITA/PA489-17/2017
RVA/Human-wt/ITA/PA367-17/2017*
RVA/Human-wt/ITA/PA23-03/2003
RVA/Human-wt/ITA/PA3-04/2004*
RVA/Human-wt/ITA/PA1-03/2003
RVA/Human-wt/ITA/PA4-03/2003*
RVA/Human-wt/ITA/PA9-04/2004
RVA/Human-wt/ITA/PA292-19/2019
RVA/Human-wt/ITA/PA306-19/2019
RVA/Human-wt/ITA/PA179-19/2019*
RVA/Human-wt/ITA/PA108-07/2007*
RVA/Human-wt/ITA/PA17-08/2008*
RVA/Human-wt/ITA/PA49-15/2015*
RVA/Human-wt/ITA/PA82-15/2015
RVA/Human-wt/ITA/PA106-15/2015
RVA/Human-wt/ITA/PA130-10/2010
RVA/Hu/USALB2764 /2005
RVA/Human-wt/ITA/PA133-11/2011
RVA/Human-wt/ITA/PA543-13/2013
RVA/Human-wt/ITA/PA83-07//linIVa3/2007*
RVA/Human-wt/ITA/PA84-08/2008*
RVA/Human-wt/ITA/PA152-12/2012
RVA/Human-wt/ITA/PA181-12/2012
RVA/Human-wt/ITA/PA188-12/2012*
RVA/Human-wt/ITA/PA208-12/2012
RVA/Vaccine/USA/RotaTeq-SC2-9/1992/G2P[75]
RVA/Human-tc/USA/DS-1-1976
G2P[4]-IVa1
G2P[4]-IVa3
89
89
86
60
84
70
86
75
100
70
72
81
88
92
0,005

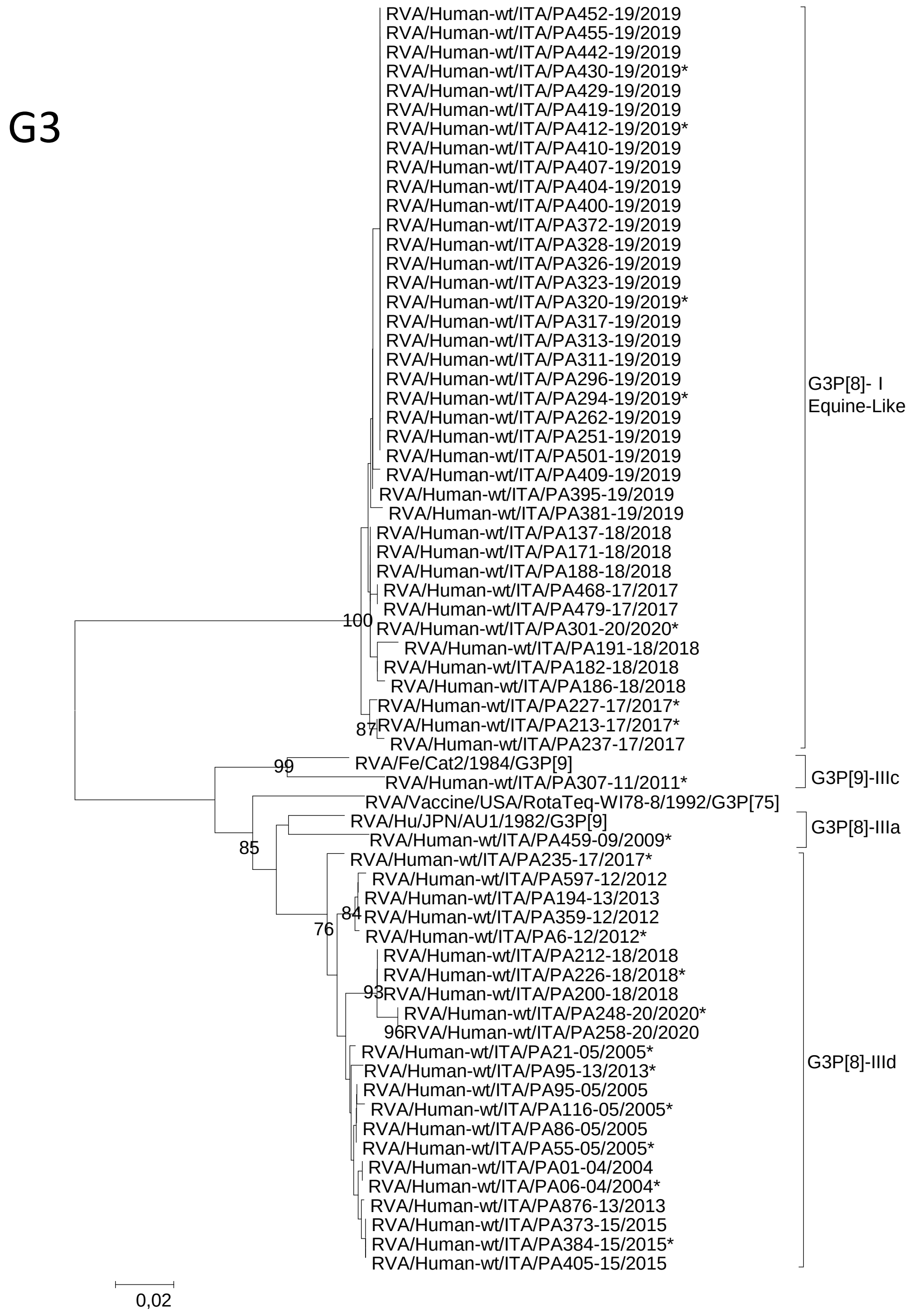
G3
RVA/Human-wt/ITA/PA452-19/2019
RVA/Human-wt/ITA/PA455-19/2019
RVA/Human-wt/ITA/PA442-19/2019
RVA/Human-wt/ITA/PA430-19/2019*
RVA/Human-wt/ITA/PA429-19/2019
RVA/Human-wt/ITA/PA419-19/2019
RVA/Human-wt/ITA/PA412-19/2019*
RVA/Human-wt/ITA/PA410-19/2019
RVA/Human-wt/ITA/PA407-19/2019
RVA/Human-wt/ITA/PA404-19/2019
RVA/Human-wt/ITA/PA400-19/2019
RVA/Human-wt/ITA/PA372-19/2019
RVA/Human-wt/ITA/PA328-19/2019
RVA/Human-wt/ITA/PA326-19/2019
RVA/Human-wt/ITA/PA323-19/2019
RVA/Human-wt/ITA/PA320-19/2019*
RVA/Human-wt/ITA/PA317-19/2019
RVA/Human-wt/ITA/PA313-19/2019
RVA/Human-wt/ITA/PA311-19/2019
RVA/Human-wt/ITA/PA296-19/2019
RVA/Human-wt/ITA/PA294-19/2019*
RVA/Human-wt/ITA/PA262-19/2019
RVA/Human-wt/ITA/PA251-19/2019
RVA/Human-wt/ITA/PA501-19/2019
RVA/Human-wt/ITA/PA409-19/2019
RVA/Human-wt/ITA/PA395-19/2019
RVA/Human-wt/ITA/PA381-19/2019
RVA/Human-wt/ITA/PA137-18/2018
RVA/Human-wt/ITA/PA171-18/2018
RVA/Human-wt/ITA/PA188-18/2018
RVA/Human-wt/ITA/PA468-17/2017
RVA/Human-wt/ITA/PA479-17/2017
RVA/Human-wt/ITA/PA301-20/2020*
RVA/Human-wt/ITA/PA191-18/2018
RVA/Human-wt/ITA/PA182-18/2018
RVA/Human-wt/ITA/PA186-18/2018
RVA/Human-wt/ITA/PA227-17/2017*
RVA/Human-wt/ITA/PA213-17/2017*
RVA/Human-wt/ITA/PA237-17/2017
RVA/Fe/Cat2/1984/G3P[9]
RVA/Human-wt/ITA/PA307-11/2011*
RVA/Vaccine/USA/RotaTeq-WI78-8/1992/G3P[75]
RVA/Hu/JPN/AU1/1982/G3P[9]
RVA/Human-wt/ITA/PA459-09/2009*
RVA/Human-wt/ITA/PA235-17/2017*
RVA/Human-wt/ITA/PA597-12/2012
RVA/Human-wt/ITA/PA194-13/2013
RVA/Human-wt/ITA/PA359-12/2012
RVA/Human-wt/ITA/PA6-12/2012*
RVA/Human-wt/ITA/PA212-18/2018
RVA/Human-wt/ITA/PA226-18/2018*
RVA/Human-wt/ITA/PA200-18/2018
RVA/Human-wt/ITA/PA248-20/2020*
RVA/Human-wt/ITA/PA258-20/2020
RVA/Human-wt/ITA/PA21-05/2005*
RVA/Human-wt/ITA/PA95-13/2013*
RVA/Human-wt/ITA/PA95-05/2005
RVA/Human-wt/ITA/PA116-05/2005*
RVA/Human-wt/ITA/PA86-05/2005
RVA/Human-wt/ITA/PA55-05/2005*
RVA/Human-wt/ITA/PA01-04/2004
RVA/Human-wt/ITA/PA06-04/2004*
RVA/Human-wt/ITA/PA876-13/2013
RVA/Human-wt/ITA/PA373-15/2015
RVA/Human-wt/ITA/PA384-15/2015*
RVA/Human-wt/ITA/PA405-15/2015
G3P[8]- I
Equine-Like
G3P[9]-IIIc
G3P[8]-IIIa
G3P[8]-IIId
100
87
99
85
84
76
93
96
0,02

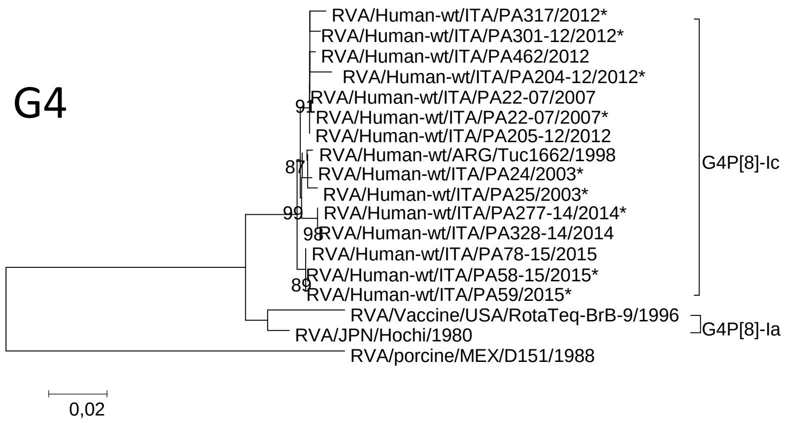
G4
RVA/Human-wt/ITA/PA317/2012*
RVA/Human-wt/ITA/PA301-12/2012*
RVA/Human-wt/ITA/PA462/2012
RVA/Human-wt/ITA/PA204-12/2012*
91
RVA/Human-wt/ITA/PA22-07/2007
RVA/Human-wt/ITA/PA22-07/2007*
RVA/Human-wt/ITA/PA205-12/2012
RVA/Human-wt/ARG/Tuc1662/1998
87
RVA/Human-wt/ITA/PA24/2003*
RVA/Human-wt/ITA/PA25/2003*
99
RVA/Human-wt/ITA/PA277-14/2014*
98
RVA/Human-wt/ITA/PA328-14/2014
RVA/Human-wt/ITA/PA78-15/2015
RVA/Human-wt/ITA/PA58-15/2015*
89
RVA/Human-wt/ITA/PA59/2015*
RVA/Vaccine/USA/RotaTeq-BrB-9/1996
RVA/JPN/Hochi/1980
RVA/porcine/MEX/D151/1988
G4P[8]-Ic
G4P[8]-Ia
0,02

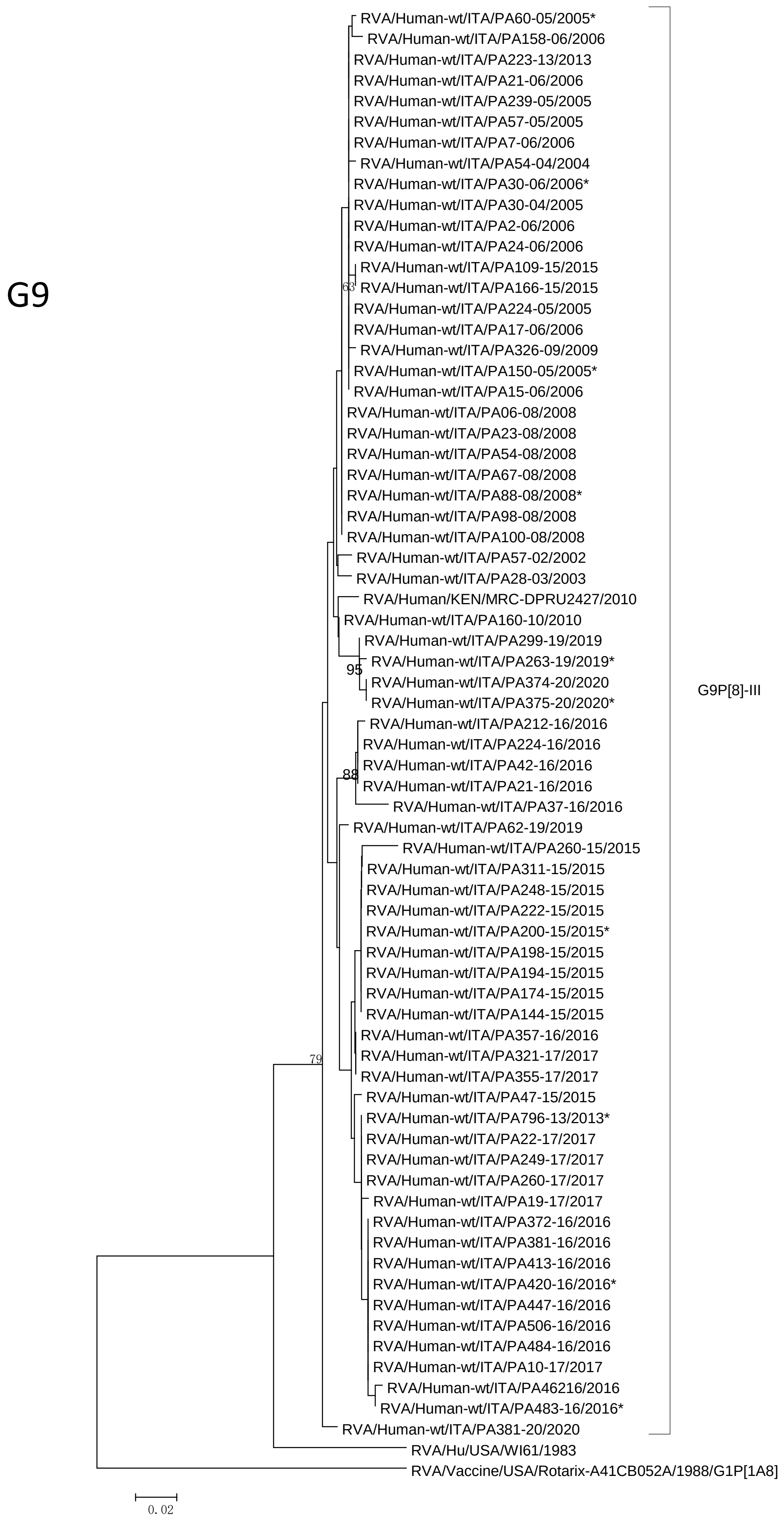
G9
RVA/Human-wt/ITA/PA60-05/2005*
RVA/Human-wt/ITA/PA158-06/2006
RVA/Human-wt/ITA/PA223-13/2013
RVA/Human-wt/ITA/PA21-06/2006
RVA/Human-wt/ITA/PA239-05/2005
RVA/Human-wt/ITA/PA57-05/2005
RVA/Human-wt/ITA/PA7-06/2006
RVA/Human-wt/ITA/PA54-04/2004
RVA/Human-wt/ITA/PA30-06/2006*
RVA/Human-wt/ITA/PA30-04/2005
RVA/Human-wt/ITA/PA2-06/2006
RVA/Human-wt/ITA/PA24-06/2006
RVA/Human-wt/ITA/PA109-15/2015
63
RVA/Human-wt/ITA/PA166-15/2015
RVA/Human-wt/ITA/PA224-05/2005
RVA/Human-wt/ITA/PA17-06/2006
RVA/Human-wt/ITA/PA326-09/2009
RVA/Human-wt/ITA/PA150-05/2005*
RVA/Human-wt/ITA/PA15-06/2006
RVA/Human-wt/ITA/PA06-08/2008
RVA/Human-wt/ITA/PA23-08/2008
RVA/Human-wt/ITA/PA54-08/2008
RVA/Human-wt/ITA/PA67-08/2008
RVA/Human-wt/ITA/PA88-08/2008*
RVA/Human-wt/ITA/PA98-08/2008
RVA/Human-wt/ITA/PA100-08/2008
RVA/Human-wt/ITA/PA57-02/2002
RVA/Human-wt/ITA/PA28-03/2003
RVA/Human/KEN/MRC-DPRU2427/2010
RVA/Human-wt/ITA/PA160-10/2010
RVA/Human-wt/ITA/PA299-19/2019
95
RVA/Human-wt/ITA/PA263-19/2019*
RVA/Human-wt/ITA/PA374-20/2020
RVA/Human-wt/ITA/PA375-20/2020*
RVA/Human-wt/ITA/PA212-16/2016
RVA/Human-wt/ITA/PA224-16/2016
RVA/Human-wt/ITA/PA42-16/2016
88
RVA/Human-wt/ITA/PA21-16/2016
RVA/Human-wt/ITA/PA37-16/2016
RVA/Human-wt/ITA/PA62-19/2019
RVA/Human-wt/ITA/PA260-15/2015
RVA/Human-wt/ITA/PA311-15/2015
RVA/Human-wt/ITA/PA248-15/2015
RVA/Human-wt/ITA/PA222-15/2015
RVA/Human-wt/ITA/PA200-15/2015*
RVA/Human-wt/ITA/PA198-15/2015
RVA/Human-wt/ITA/PA194-15/2015
RVA/Human-wt/ITA/PA174-15/2015
RVA/Human-wt/ITA/PA144-15/2015
RVA/Human-wt/ITA/PA357-16/2016
79
RVA/Human-wt/ITA/PA321-17/2017
RVA/Human-wt/ITA/PA355-17/2017
RVA/Human-wt/ITA/PA47-15/2015
RVA/Human-wt/ITA/PA796-13/2013*
RVA/Human-wt/ITA/PA22-17/2017
RVA/Human-wt/ITA/PA249-17/2017
RVA/Human-wt/ITA/PA260-17/2017
RVA/Human-wt/ITA/PA19-17/2017
RVA/Human-wt/ITA/PA372-16/2016
RVA/Human-wt/ITA/PA381-16/2016
RVA/Human-wt/ITA/PA413-16/2016
RVA/Human-wt/ITA/PA420-16/2016*
RVA/Human-wt/ITA/PA447-16/2016
RVA/Human-wt/ITA/PA506-16/2016
RVA/Human-wt/ITA/PA484-16/2016
RVA/Human-wt/ITA/PA10-17/2017
RVA/Human-wt/ITA/PA46216/2016
RVA/Human-wt/ITA/PA483-16/2016*
RVA/Human-wt/ITA/PA381-20/2020
RVA/Hu/USA/WI61/1983
RVA/Vaccine/USA/Rotarix-A41CB052A/1988/G1P[1A8]
G9P[8]-III
0. 02

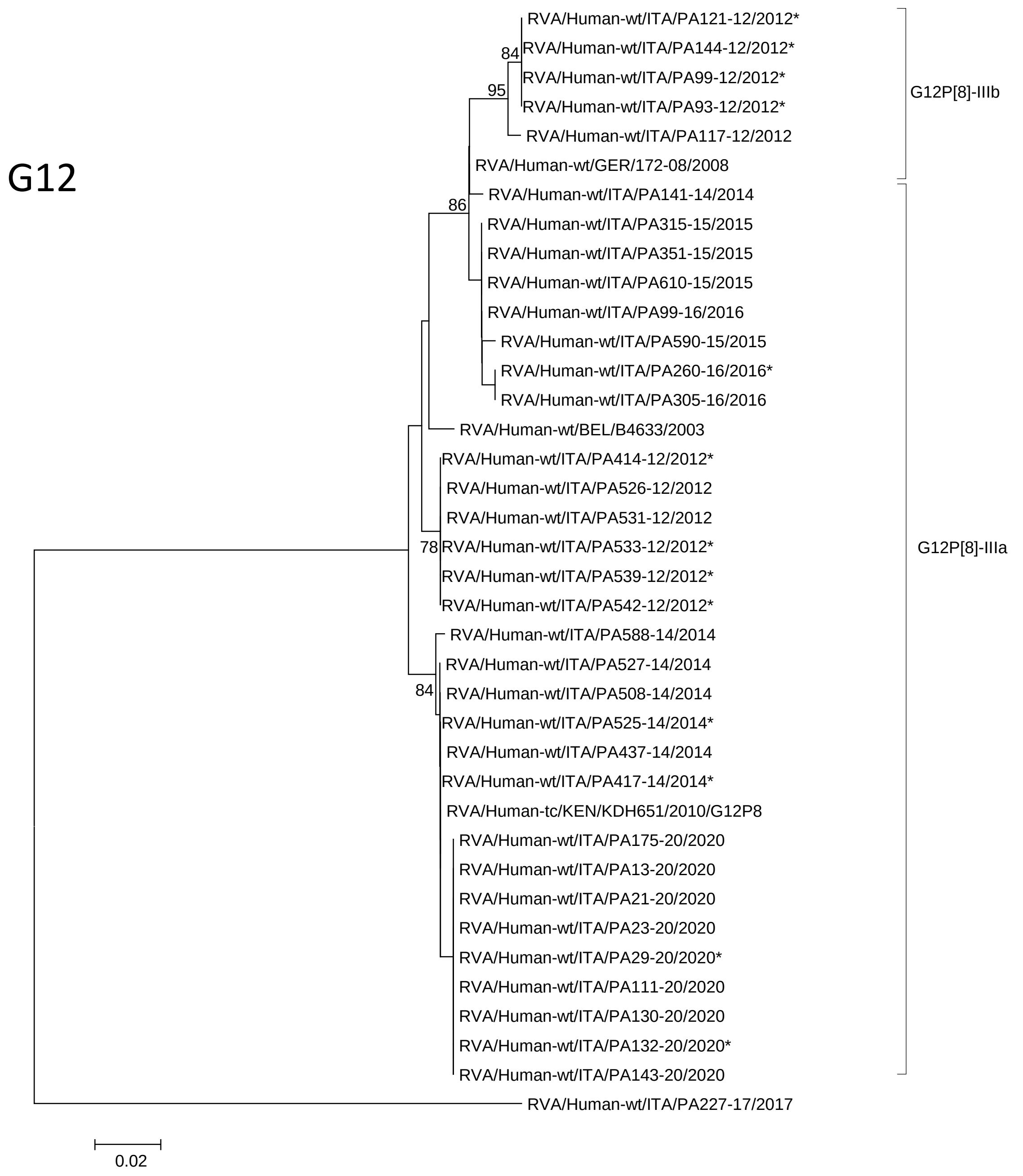
G12
RVA/Human-wt/ITA/PA121-12/2012*
RVA/Human-wt/ITA/PA144-12/2012*
RVA/Human-wt/ITA/PA99-12/2012*
RVA/Human-wt/ITA/PA93-12/2012*
RVA/Human-wt/ITA/PA117-12/2012
RVA/Human-wt/GER/172-08/2008
RVA/Human-wt/ITA/PA141-14/2014
RVA/Human-wt/ITA/PA315-15/2015
RVA/Human-wt/ITA/PA351-15/2015
RVA/Human-wt/ITA/PA610-15/2015
RVA/Human-wt/ITA/PA99-16/2016
RVA/Human-wt/ITA/PA590-15/2015
RVA/Human-wt/ITA/PA260-16/2016*
RVA/Human-wt/ITA/PA305-16/2016
RVA/Human-wt/BEL/B4633/2003
RVA/Human-wt/ITA/PA414-12/2012*
RVA/Human-wt/ITA/PA526-12/2012
RVA/Human-wt/ITA/PA531-12/2012
RVA/Human-wt/ITA/PA533-12/2012*
RVA/Human-wt/ITA/PA539-12/2012*
RVA/Human-wt/ITA/PA542-12/2012*
RVA/Human-wt/ITA/PA588-14/2014
RVA/Human-wt/ITA/PA527-14/2014
RVA/Human-wt/ITA/PA508-14/2014
RVA/Human-wt/ITA/PA525-14/2014*
RVA/Human-wt/ITA/PA437-14/2014
RVA/Human-wt/ITA/PA417-14/2014*
RVA/Human-tc/KEN/KDH651/2010/G12P8
RVA/Human-wt/ITA/PA175-20/2020
RVA/Human-wt/ITA/PA13-20/2020
RVA/Human-wt/ITA/PA21-20/2020
RVA/Human-wt/ITA/PA23-20/2020
RVA/Human-wt/ITA/PA29-20/2020*
RVA/Human-wt/ITA/PA111-20/2020
RVA/Human-wt/ITA/PA130-20/2020
RVA/Human-wt/ITA/PA132-20/2020*
RVA/Human-wt/ITA/PA143-20/2020
RVA/Human-wt/ITA/PA227-17/2017
84
95
86
78
84
G12P[8]-IIIb
G12P[8]-IIIa
0.02

P[4]

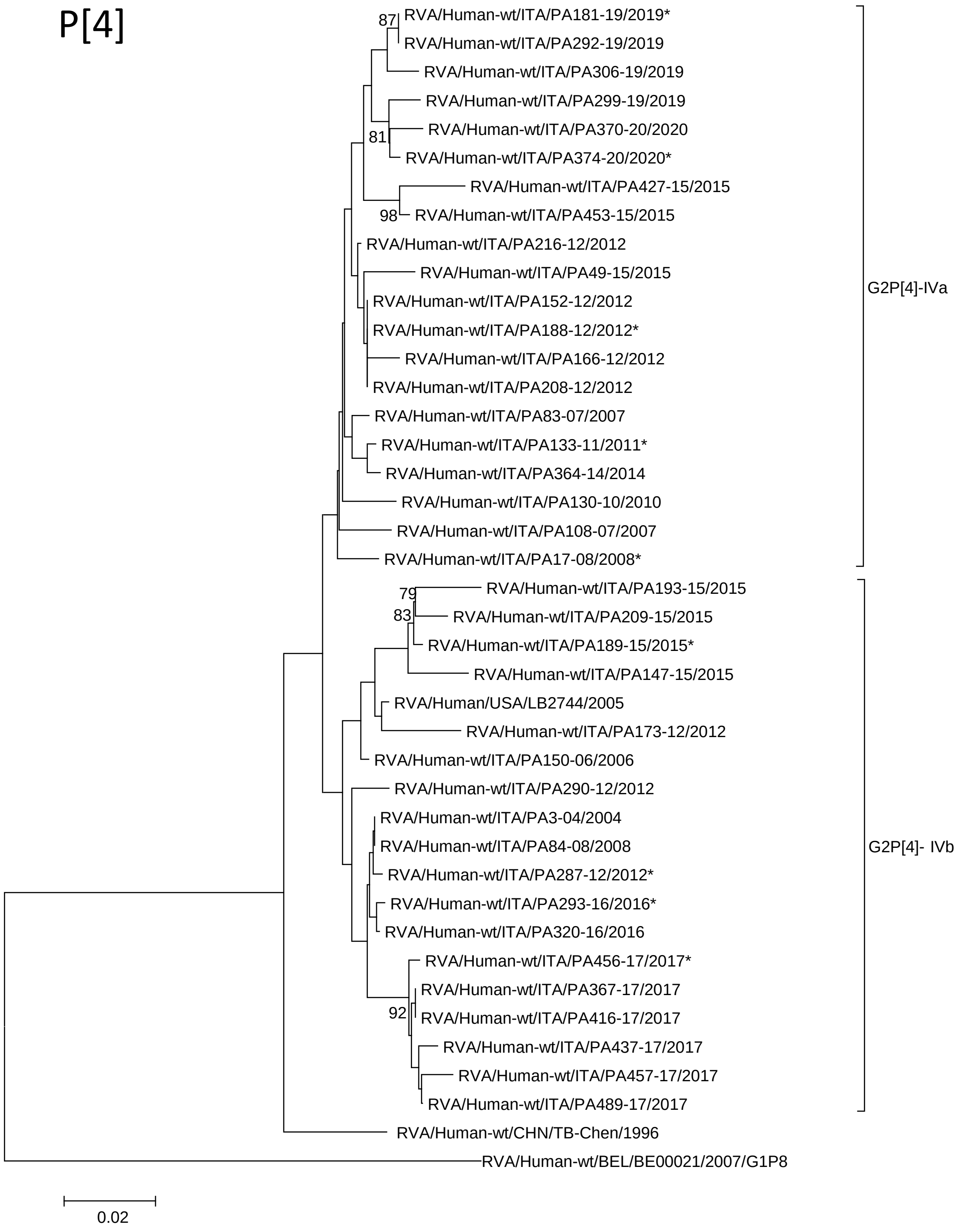
87
RVA/Human-wt/ITA/PA181-19/2019*
RVA/Human-wt/ITA/PA292-19/2019
RVA/Human-wt/ITA/PA306-19/2019
RVA/Human-wt/ITA/PA299-19/2019
81
RVA/Human-wt/ITA/PA370-20/2020
RVA/Human-wt/ITA/PA374-20/2020*
RVA/Human-wt/ITA/PA427-15/2015
98
RVA/Human-wt/ITA/PA453-15/2015
RVA/Human-wt/ITA/PA216-12/2012
RVA/Human-wt/ITA/PA49-15/2015
RVA/Human-wt/ITA/PA152-12/2012
RVA/Human-wt/ITA/PA188-12/2012*
RVA/Human-wt/ITA/PA166-12/2012
RVA/Human-wt/ITA/PA208-12/2012
RVA/Human-wt/ITA/PA83-07/2007
RVA/Human-wt/ITA/PA133-11/2011*
RVA/Human-wt/ITA/PA364-14/2014
RVA/Human-wt/ITA/PA130-10/2010
RVA/Human-wt/ITA/PA108-07/2007
RVA/Human-wt/ITA/PA17-08/2008*
G2P[4]-IVa
79
RVA/Human-wt/ITA/PA193-15/2015
83
RVA/Human-wt/ITA/PA209-15/2015
RVA/Human-wt/ITA/PA189-15/2015*
RVA/Human-wt/ITA/PA147-15/2015
RVA/Human/USA/LB2744/2005
RVA/Human-wt/ITA/PA173-12/2012
RVA/Human-wt/ITA/PA150-06/2006
RVA/Human-wt/ITA/PA290-12/2012
RVA/Human-wt/ITA/PA3-04/2004
RVA/Human-wt/ITA/PA84-08/2008
RVA/Human-wt/ITA/PA287-12/2012*
RVA/Human-wt/ITA/PA293-16/2016*
RVA/Human-wt/ITA/PA320-16/2016
RVA/Human-wt/ITA/PA456-17/2017*
RVA/Human-wt/ITA/PA367-17/2017
92
RVA/Human-wt/ITA/PA416-17/2017
RVA/Human-wt/ITA/PA437-17/2017
RVA/Human-wt/ITA/PA457-17/2017
RVA/Human-wt/ITA/PA489-17/2017
G2P[4]- IVb
RVA/Human-wt/CHN/TB-Chen/1996
RVA/Human-wt/BEL/BE00021/2007/G1P8
0.02

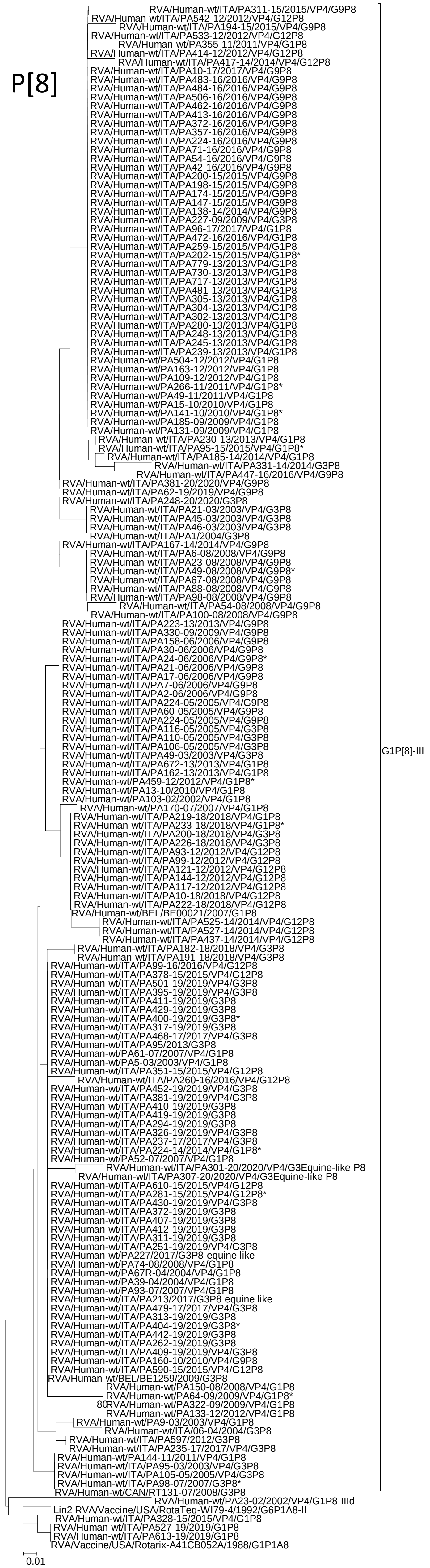
P[8]
RVA/Human-wt/ITA/PA311-15/2015/VP4/G9P8
RVA/Human-wt/ITA/PA542-12/2012/VP4/G12P8
RVA/Human-wt/ITA/PA194-15/2015/VP4/G9P8
RVA/Human-wt/ITA/PA533-12/2012/VP4/G12P8
RVA/Human-wt/PA355-11/2011/VP4/G1P8
RVA/Human-wt/ITA/PA414-12/2012/VP4/G12P8
RVA/Human-wt/ITA/PA417-14/2014/VP4/G12P8
RVA/Human-wt/ITA/PA10-17/2017/VP4/G9P8
RVA/Human-wt/ITA/PA483-16/2016/VP4/G9P8
RVA/Human-wt/ITA/PA484-16/2016/VP4/G9P8
RVA/Human-wt/ITA/PA506-16/2016/VP4/G9P8
RVA/Human-wt/ITA/PA462-16/2016/VP4/G9P8
RVA/Human-wt/ITA/PA413-16/2016/VP4/G9P8
RVA/Human-wt/ITA/PA372-16/2016/VP4/G9P8
RVA/Human-wt/ITA/PA357-16/2016/VP4/G9P8
RVA/Human-wt/ITA/PA224-16/2016/VP4/G9P8
RVA/Human-wt/ITA/PA71-16/2016/VP4/G9P8
RVA/Human-wt/ITA/PA54-16/2016/VP4/G9P8
RVA/Human-wt/ITA/PA42-16/2016/VP4/G9P8
RVA/Human-wt/ITA/PA200-15/2015/VP4/G9P8
RVA/Human-wt/ITA/PA198-15/2015/VP4/G9P8
RVA/Human-wt/ITA/PA174-15/2015/VP4/G9P8
RVA/Human-wt/ITA/PA147-15/2015/VP4/G9P8
RVA/Human-wt/ITA/PA138-14/2014/VP4/G9P8
RVA/Human-wt/ITA/PA227-09/2009/VP4/G3P8
RVA/Human-wt/ITA/PA96-17/2017/VP4/G1P8
RVA/Human-wt/ITA/PA472-16/2016/VP4/G1P8
RVA/Human-wt/ITA/PA259-15/2015/VP4/G1P8
RVA/Human-wt/ITA/PA202-15/2015/VP4/G1P8*
RVA/Human-wt/ITA/PA779-13/2013/VP4/G1P8
RVA/Human-wt/ITA/PA730-13/2013/VP4/G1P8
RVA/Human-wt/ITA/PA717-13/2013/VP4/G1P8
RVA/Human-wt/ITA/PA481-13/2013/VP4/G1P8
RVA/Human-wt/ITA/PA305-13/2013/VP4/G1P8
RVA/Human-wt/ITA/PA304-13/2013/VP4/G1P8
RVA/Human-wt/ITA/PA302-13/2013/VP4/G1P8
RVA/Human-wt/ITA/PA280-13/2013/VP4/G1P8
RVA/Human-wt/ITA/PA248-13/2013/VP4/G1P8
RVA/Human-wt/ITA/PA245-13/2013/VP4/G1P8
RVA/Human-wt/ITA/PA239-13/2013/VP4/G1P8
RVA/Human-wt/PA504-12/2012/VP4/G1P8
RVA/Human-wt/PA163-12/2012/VP4/G1P8
RVA/Human-wt/PA109-12/2012/VP4/G1P8
RVA/Human-wt/PA266-11/2011/VP4/G1P8*
RVA/Human-wt/PA49-11/2011/VP4/G1P8
RVA/Human-wt/PA15-10/2010/VP4/G1P8
RVA/Human-wt/PA141-10/2010/VP4/G1P8*
RVA/Human-wt/PA185-09/2009/VP4/G1P8
RVA/Human-wt/PA131-09/2009/VP4/G1P8
RVA/Human-wt/ITA/PA230-13/2013/VP4/G1P8
RVA/Human-wt/ITA/PA95-15/2015/VP4/G1P8*
RVA/Human-wt/ITA/PA185-14/2014/VP4/G1P8
RVA/Human-wt/ITA/PA331-14/2014/G3P8
RVA/Human-wt/ITA/PA447-16/2016/VP4/G9P8
RVA/Human-wt/ITA/PA381-20/2020/VP4/G9P8
RVA/Human-wt/ITA/PA62-19/2019/VP4/G9P8
RVA/Human-wt/ITA/PA248-20/2020/G3P8
RVA/Human-wt/ITA/PA21-03/2003/VP4/G3P8
RVA/Human-wt/ITA/PA45-03/2003/VP4/G3P8
RVA/Human-wt/ITA/PA46-03/2003/VP4/G3P8
RVA/Human-wt/ITA/PA1/2004/G3P8
RVA/Human-wt/ITA/PA167-14/2014/VP4/G9P8
RVA/Human-wt/ITA/PA6-08/2008/VP4/G9P8
RVA/Human-wt/ITA/PA23-08/2008/VP4/G9P8
RVA/Human-wt/ITA/PA49-08/2008/VP4/G9P8*
RVA/Human-wt/ITA/PA67-08/2008/VP4/G9P8
RVA/Human-wt/ITA/PA88-08/2008/VP4/G9P8
RVA/Human-wt/ITA/PA98-08/2008/VP4/G9P8
RVA/Human-wt/ITA/PA54-08/2008/VP4/G9P8
RVA/Human-wt/ITA/PA100-08/2008/VP4/G9P8
RVA/Human-wt/ITA/PA223-13/2013/VP4/G9P8
RVA/Human-wt/ITA/PA330-09/2009/VP4/G9P8
RVA/Human-wt/ITA/PA158-06/2006/VP4/G9P8
RVA/Human-wt/ITA/PA30-06/2006/VP4/G9P8
RVA/Human-wt/ITA/PA24-06/2006/VP4/G9P8*
RVA/Human-wt/ITA/PA21-06/2006/VP4/G9P8
RVA/Human-wt/ITA/PA17-06/2006/VP4/G9P8
RVA/Human-wt/ITA/PA7-06/2006/VP4/G9P8
RVA/Human-wt/ITA/PA2-06/2006/VP4/G9P8
RVA/Human-wt/ITA/PA224-05/2005/VP4/G9P8
RVA/Human-wt/ITA/PA60-05/2005/VP4/G9P8
RVA/Human-wt/ITA/PA224-05/2005/VP4/G9P8
RVA/Human-wt/ITA/PA116-05/2005/VP4/G3P8
RVA/Human-wt/ITA/PA110-05/2005/VP4/G3P8
RVA/Human-wt/ITA/PA106-05/2005/VP4/G3P8
RVA/Human-wt/ITA/PA49-03/2003/VP4/G3P8
RVA/Human-wt/ITA/PA672-13/2013/VP4/G1P8
RVA/Human-wt/ITA/PA162-13/2013/VP4/G1P8
RVA/Human-wt/PA459-12/2012/VP4/G1P8*
RVA/Human-wt/PA13-10/2010/VP4/G1P8
RVA/Human-wt/PA103-02/2002/VP4/G1P8
RVA/Human-wt/PA170-07/2007/VP4/G1P8
RVA/Human-wt/ITA/PA219-18/2018/VP4/G1P8
RVA/Human-wt/ITA/PA233-18/2018/VP4/G1P8*
RVA/Human-wt/ITA/PA200-18/2018/VP4/G3P8
RVA/Human-wt/ITA/PA226-18/2018/VP4/G3P8
RVA/Human-wt/ITA/PA93-12/2012/VP4/G12P8
RVA/Human-wt/ITA/PA99-12/2012/VP4/G12P8
RVA/Human-wt/ITA/PA121-12/2012/VP4/G12P8
RVA/Human-wt/ITA/PA144-12/2012/VP4/G12P8
RVA/Human-wt/ITA/PA117-12/2012/VP4/G12P8
RVA/Human-wt/ITA/PA10-18/2018/VP4/G12P8
RVA/Human-wt/ITA/PA222-18/2018/VP4/G12P8
RVA/Human-wt/BEL/BE00021/2007/G1P8
RVA/Human-wt/ITA/PA525-14/2014/VP4/G12P8
RVA/Human-wt/ITA/PA527-14/2014/VP4/G12P8
RVA/Human-wt/ITA/PA437-14/2014/VP4/G12P8
RVA/Human-wt/ITA/PA182-18/2018/VP4/G3P8
RVA/Human-wt/ITA/PA191-18/2018/VP4/G3P8
RVA/Human-wt/ITA/PA99-16/2016/VP4/G12P8
RVA/Human-wt/ITA/PA378-15/2015/VP4/G12P8
RVA/Human-wt/ITA/PA501-19/2019/VP4/G3P8
RVA/Human-wt/ITA/PA395-19/2019/VP4/G3P8
RVA/Human-wt/ITA/PA411-19/2019/G3P8
RVA/Human-wt/ITA/PA429-19/2019/G3P8
RVA/Human-wt/ITA/PA400-19/2019/G3P8*
RVA/Human-wt/ITA/PA317-19/2019/G3P8
RVA/Human-wt/ITA/PA468-17/2017/VP4/G3P8
RVA/Human-wt/ITA/PA95/2013/G3P8
RVA/Human-wt/PA61-07/2007/VP4/G1P8
RVA/Human-wt/PA5-03/2003/VP4/G1P8
RVA/Human-wt/ITA/PA351-15/2015/VP4/G12P8
RVA/Human-wt/ITA/PA260-16/2016/VP4/G12P8
RVA/Human-wt/ITA/PA452-19/2019/VP4/G3P8
RVA/Human-wt/ITA/PA381-19/2019/VP4/G3P8
RVA/Human-wt/ITA/PA410-19/2019/G3P8
RVA/Human-wt/ITA/PA419-19/2019/G3P8
RVA/Human-wt/ITA/PA294-19/2019/G3P8
RVA/Human-wt/ITA/PA326-19/2019/VP4/G3P8
RVA/Human-wt/ITA/PA237-17/2017/VP4/G3P8
RVA/Human-wt/ITA/PA224-14/2014/VP4/G1P8*
RVA/Human-wt/PA52-07/2007/VP4/G1P8
RVA/Human-wt/ITA/PA301-20/2020/VP4/G3Equine-like P8
RVA/Human-wt/ITA/PA307-20/2020/VP4/G3Equine-like P8
RVA/Human-wt/ITA/PA610-15/2015/VP4/G12P8
RVA/Human-wt/ITA/PA281-15/2015/VP4/G12P8*
RVA/Human-wt/ITA/PA430-19/2019/VP4/G3P8
RVA/Human-wt/ITA/PA372-19/2019/G3P8
RVA/Human-wt/ITA/PA407-19/2019/G3P8
RVA/Human-wt/ITA/PA412-19/2019/G3P8
RVA/Human-wt/ITA/PA311-19/2019/G3P8
RVA/Human-wt/ITA/PA251-19/2019/VP4/G3P8
RVA/Human-wt/PA227/2017/G3P8 equine like
RVA/Human-wt/PA74-08/2008/VP4/G1P8
RVA/Human-wt/PA67R-04/2004/VP4/G1P8
RVA/Human-wt/PA39-04/2004/VP4/G1P8
RVA/Human-wt/PA93-07/2007/VP4/G1P8
RVA/Human-wt/ITA/PA213/2017/G3P8 equine like
RVA/Human-wt/ITA/PA479-17/2017/VP4/G3P8
RVA/Human-wt/ITA/PA313-19/2019/G3P8
RVA/Human-wt/ITA/PA404-19/2019/G3P8*
RVA/Human-wt/ITA/PA442-19/2019/G3P8
RVA/Human-wt/ITA/PA262-19/2019/G3P8
RVA/Human-wt/ITA/PA409-19/2019/VP4/G3P8
RVA/Human-wt/ITA/PA160-10/2010/VP4/G9P8
RVA/Human-wt/ITA/PA590-15/2015/VP4/G12P8
RVA/Human-wt/BEL/BE1259/2009/G3P8
RVA/Human-wt/PA150-08/2008/VP4/G1P8
RVA/Human-wt/PA64-09/2009/VP4/G1P8*
80
RVA/Human-wt/PA322-09/2009/VP4/G1P8
RVA/Human-wt/PA133-12/2012/VP4/G1P8
RVA/Human-wt/PA9-03/2003/VP4/G1P8
RVA/Human-wt/ITA/06-04/2004/G3P8
RVA/Human-wt/ITA/PA597/2012/G3P8
RVA/Human-wt/ITA/PA235-17/2017/VP4/G3P8
RVA/Human-wt/PA144-11/2011/VP4/G1P8
RVA/Human-wt/ITA/PA95-03/2003/VP4/G3P8
RVA/Human-wt/ITA/PA105-05/2005/VP4/G3P8
RVA/Human-wt/ITA/PA98-07/2007/G3P8*
RVA/Human-wt/CAN/RT131-07/2008/G3P8
G1P[8]-III
RVA/Human-wt/PA23-02/2002/VP4/G1P8 IIId
Lin2 RVA/Vaccine/USA/RotaTeq-WI79-4/1992/G6P1A8-II
RVA/Human-wt/ITA/PA328-15/2015/VP4/G1P8
RVA/Human-wt/ITA/PA527-19/2019/G1P8
RVA/Human-wt/ITA/PA613-19/2019/G1P8
RVA/Vaccine/USA/Rotarix-A41CB052A/1988/G1P1A8
0.01

Figure S1: Phylogenetic analysis of partial VP7 (G1-4, G9 and G12) and VP4 (P[4]-P[8]) nucleotide sequences of Italian strains detected in the study. The phylogenetic trees were built using the neighbor-joining method and Kimura’ s two-parameter model, and bootstrapped with 1000 repetitions. Bootstrap values 75% are indicated. * Sequences availables in GenBank
